# Silent Sinus Syndrome in a Paediatric Patient: An Incidental Diagnosis Following Facial Trauma

**DOI:** 10.7759/cureus.107681

**Published:** 2026-04-24

**Authors:** Eilidh Thomas, Daniel J Shepherd, Simon Thorne, James Sloane

**Affiliations:** 1 Oral and Maxillofacial Surgery, Royal Surrey County Hospital, Guildford, GBR; 2 Otolaryngology, Royal Surrey County Hospital, Guildford, GBR

**Keywords:** enophthalmos, facial asymmetry, facial trauma, functional endoscopic sinus surgery, incidental finding, maxillary sinus atelectasis, paediatric, silent sinus syndrome

## Abstract

Silent sinus syndrome (SSS) is a rare condition characterised by spontaneous unilateral atelectasis of a paranasal sinus, most commonly the maxillary sinus, typically presenting in adults with progressive enophthalmos and hypoglobus. Paediatric cases are uncommon and may present subtly, often lacking the classic features observed in adults.

We report an incidental diagnosis of SSS in a 13-year-old boy following minor facial trauma. Clinical assessment raised the concern for a facial fracture due to subtle malar asymmetry. Plain radiographs demonstrated right maxillary sinus opacification with associated malar asymmetry, without obvious fracture, prompting further imaging. Computed tomography excluded an acute fracture but revealed changes consistent with SSS. The patient underwent functional endoscopic sinus surgery (FESS) to prevent disease progression.

This case highlights the importance of considering SSS in trauma presentations where clinical findings and radiographic appearances are incongruent. Early recognition is essential to prevent progressive facial deformity and potential visual complications, particularly in the paediatric population.

## Introduction

Silent sinus syndrome (SSS) is a rare condition characterised by spontaneous unilateral atelectasis of a paranasal sinus, most commonly the maxillary sinus, occurring in the absence of trauma or previous sinonasal disease [1,2]. It typically presents in adults in the third to fifth decades of life, with paediatric cases being very uncommon [2,3]. True prevalence is unknown, with evidence limited to case reports and small series. The condition is thought to arise from the obstruction of maxillary sinus drainage, resulting in chronic negative pressure, sinus collapse and progressive bony remodelling [1,4]. SSS is considered part of the spectrum of chronic maxillary atelectasis but is distinguished by the absence of preceding sinonasal symptoms or identifiable precipitating pathology. Diagnosis is based on a combination of clinical and radiological findings, including unilateral maxillary sinus opacification, inward retraction of the sinus walls and inferior displacement of the orbital floor.

The condition is often subclinical, and diagnosis is frequently delayed until progressive maxillary remodelling leads to enophthalmos and hypoglobus secondary to orbital floor descent. Patients therefore present later in the disease course, often to ophthalmology services with visual disturbance. Radiological features include a hypoplastic maxilla with sinus opacification or collapse [1,2,5]. In children, presentation may be even more subtle, increasing the likelihood of incidental or delayed diagnosis.

Given the rarity of the condition, and its particularly uncommon presentation in children, we report an incidental diagnosis in a 13-year-old boy who presented to the emergency department following minor facial trauma sustained during a football match. This case highlights the importance of recognising discordance between clinical findings and imaging in the trauma setting, prompting consideration of alternative diagnoses such as SSS.

## Case presentation

**Figure 1:**
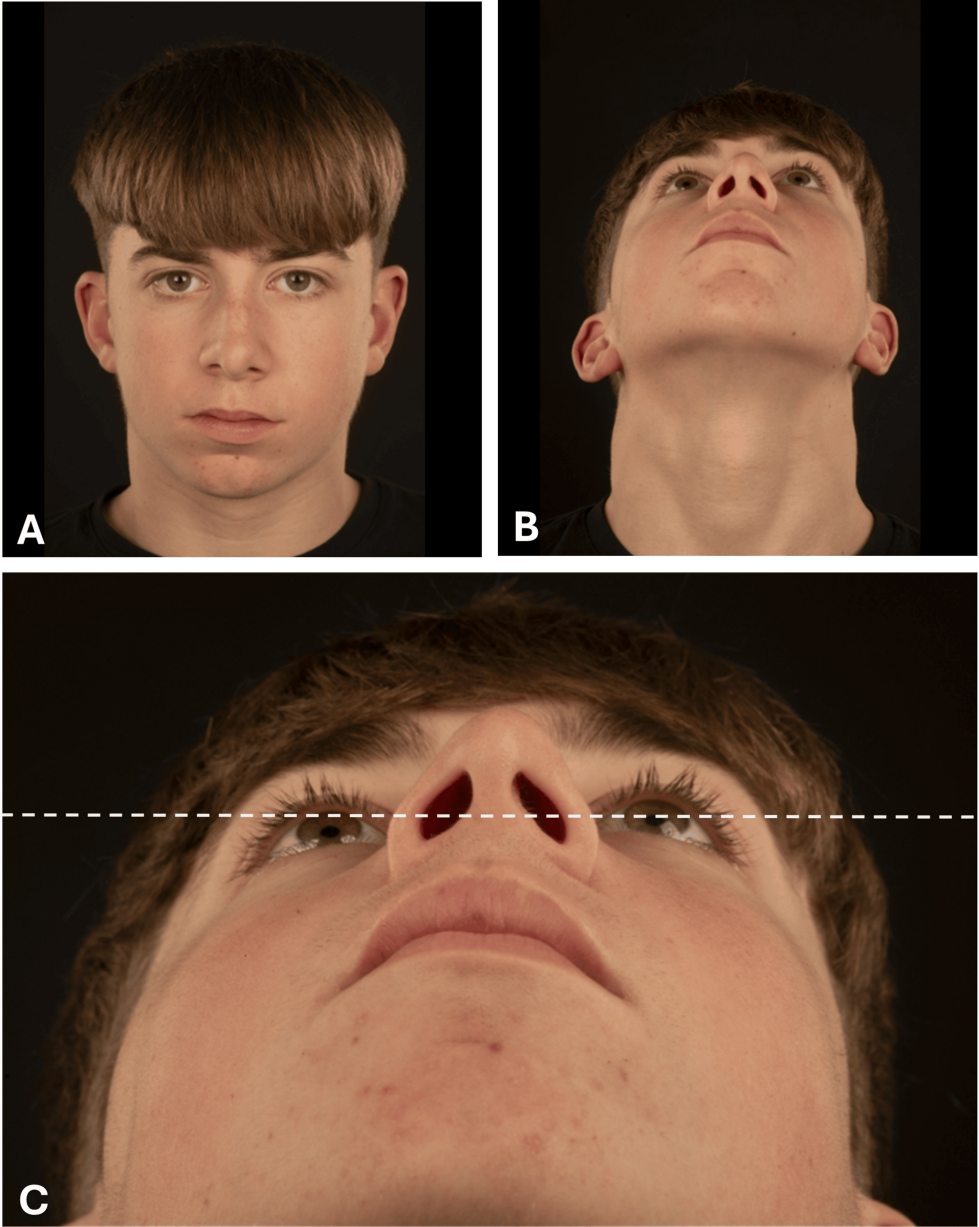
Clinical images demonstrating subtle right-sided facial asymmetry with mild malar flattening (A: frontal view, B: inferior/worm’s-eye view and C: close-up inferior view demonstrating relative depression of the right malar region). The dashed line represents a horizontal reference aligned with the left pupil for comparison. Written informed consent for publication of clinical information and images, including identifiable features, was obtained from the patient and his parents/guardian.

A 13-year-old boy presented to the emergency department following a football-related injury, having sustained a facial laceration from a kick to the right side of his face. After clearance from a head injury perspective, he was referred to the Oral and Maxillofacial Surgery team for further assessment. The impact was described as a glancing blow. Despite this, subtle but clinically discernible facial asymmetry was noted, without associated bony tenderness or ocular signs. Examination identified a right infraorbital partial thickness laceration with mild flattening of the right malar region (Figure 1), alongside normal sensation, visual acuity, pupil reactivity and full extraocular movements. Neither the patient nor his parents reported any pre-existing facial asymmetry, and there was no history of sinonasal or ocular symptoms.

**Figure 2:**
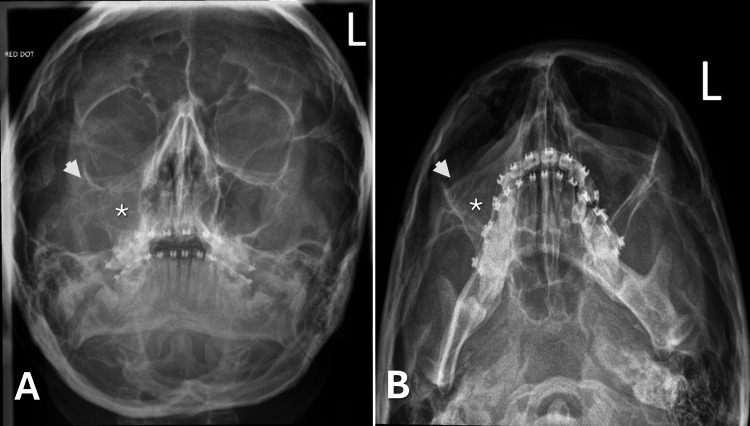
Occipitomental radiographs (A: 0° and B: 30°) demonstrating right maxillary sinus opacification and asymmetry of the right malar contour relative to the left. The asterisk indicates the right maxillary sinus. The arrow indicates the right malar contour.

Given the malar flattening in the context of facial trauma, a right zygomatic fracture was considered and radiographic imaging was undertaken. Initial plain facial bone radiographs demonstrated no obvious steps/fractures of the right zygoma and infraorbital margin; however, there was opacification of the right maxillary sinus with notable asymmetry of the malar contour (Figure 2).

**Figure 3:**
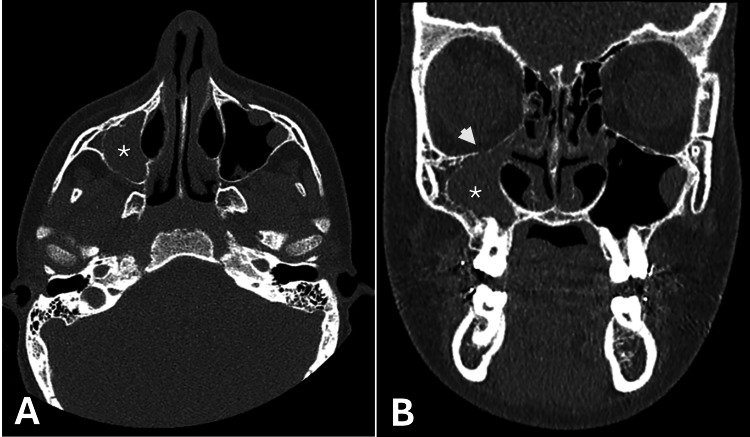
Cross-sectional imaging of facial bones (A: axial, B: coronal) demonstrating right maxillary sinus opacification with reduced volume and bony remodelling. Subtle descent of the right orbital floor in comparison to the left is best appreciated on the coronal view (B). Asterisk (A, B) indicates the right maxillary sinus. Arrow (B) indicates the right orbital floor.

In the context of the mechanism of injury, sinus opacification and unexplained facial asymmetry, computer tomography (CT) was performed to further assess for underlying facial injury and guide management. This demonstrated no acute facial bone fracture but revealed right maxillary cortical thickening and complete opacification of the right maxillary sinus, consistent with a chronic process. There was also a subtle descent of the orbital floor compared to the contralateral side (Figure 3).

In the absence of acute fracture or previous sinonasal disease, the combination of complete maxillary sinus opacification, bony remodelling with reduced sinus volume, and inferior displacement of the orbital floor was diagnostic of SSS. The superficial soft tissue trauma acted as a red herring, prompting further investigation and subsequent diagnosis. The facial laceration was closed under local anaesthesia, and the patient was referred to the Otolaryngology service for outpatient review.

On review by Otolaryngology, the patient remained asymptomatic from a sinonasal perspective, with no relevant family history. Given the risk of progressive maxillary hypoplasia during adolescence, with potential development of hypoglobus and diplopia, a decision was made to proceed with functional endoscopic sinus surgery (FESS) to prevent further disease progression.

Intraoperatively, the maxillary sinus was found to contain a large volume of thick mucoid secretions, which were evacuated. A right uncinectomy and middle meatal antrostomy were performed, and a middle meatal spacer was inserted. There were no intraoperative complications. Postoperative review at one week was unremarkable, and the patient remains under routine Otolaryngology follow-up.

## Discussion

Facial flattening and malar depression are well-recognised clinical features of midface fractures and commonly prompt suspicion of zygomatic injury in the trauma setting [6]. In this case, subtle malar flattening following facial trauma led appropriately to investigation for fracture. However, the incidental finding of an opacified maxillary sinus on plain radiographs in the absence of bony steps, tenderness or infraorbital paraesthesia introduced diagnostic uncertainty and highlights how SSS may clinically be differentiated from an acute fracture. Cross-sectional imaging is the gold standard for diagnosing both facial fractures and SSS. In the context of SSS, CT is often performed incidentally for unrelated indications [5]. In the absence of CT imaging, this presentation may have been misattributed to traumatic injury alone.

The diagnosis of SSS is based on a combination of clinical and radiological findings, including unilateral maxillary sinus opacification, inward retraction of the sinus walls, and inferior displacement of the orbital floor [1,2,5]. In the absence of acute fracture or previous sinonasal disease, these features are considered diagnostic. In this case, the absence of fracture alongside complete sinus opacification, bony remodelling and early orbital floor descent supported the diagnosis, with the superficial soft tissue injury acting as a red herring.

SSS is believed to be underdiagnosed in the paediatric population [2]. The slow progression of sinus collapse and maxillary hypoplasia may result in subtle facial asymmetry that is easily overlooked by patients and caregivers [5]. This may explain why most cases present later in adulthood, once progressive enophthalmos, hypoglobus or visual disturbance becomes clinically apparent. In children, early recognition is particularly important, given the potential for ongoing facial growth to exacerbate deformity.

The primary aim of management is to prevent further progression of facial deformity by restoring ventilation of the affected sinus [1]. FESS, consisting of uncinectomy and middle meatal antrostomy, is considered first-line treatment [1]. In our patient, copious mucoid secretions were encountered intraoperatively, consistent with chronic obstruction. Whether this reflects primary mucus accumulation or an underlying anatomical drainage abnormality remains uncertain; however, relief of obstruction remains the key therapeutic goal.

Following FESS, resolution of negative sinus pressure allows gradual expansion of the maxillary sinus and orbital volume through bony remodelling [7,8]. Several studies have demonstrated improvement in orbital floor position following sinus surgery alone, without the need for primary orbital reconstruction. As such, orbital floor reconstruction is generally reserved for patients with persistent diplopia or significant residual deformity after sinus reaeration [1,7,8]. The management strategy adopted in this case is therefore consistent with current evidence.

Although rare cases of spontaneous resolution have been described in paediatric patients, the risk of progressive facial asymmetry and visual complications in untreated disease supports early surgical intervention [9]. Given the uncertainty surrounding disease aetiology and progression, restoration of sinus ventilation remains the most reliable means of preventing long-term sequelae.

## Conclusions

This case illustrates how SSS may mimic facial fracture following minor trauma, particularly in the presence of subtle facial asymmetry. Awareness of this condition is important when clinical findings and radiographic appearances are incongruent, and should prompt consideration of alternative diagnoses and further cross-sectional imaging. Early recognition and appropriate multidisciplinary management are key to preventing progressive facial deformity and potential visual complications, particularly in paediatric patients.
